# Supplementary material for: Multi-omic responses to acute exercise in abdominal subcutaneous adipose tissue of sedentary adults: findings from MoTrPAC
Source: bioRxiv. 2026 Mar 19:2026.03.05.702363. Preprint. [Version 2] doi: 10.64898/2026.03.05.702363 (PMC13015483; doi:10.64898/2026.03.05.702363)
Supplement: Supplement 7 [file NIHPP2026.03.05.702363v2-supplement-7.pdf]

bioRxiv preprint doi: https://doi.org/10.64898/2026.03.05.702363; this version posted March 19, 2026. The copyright holder for this preprint (which was not certified by peer review) is the author/funder, who has granted bioRxiv a license to display the preprint in perpetuity. It is made available under aCC-BY 4.0 International license.

Fig S1A

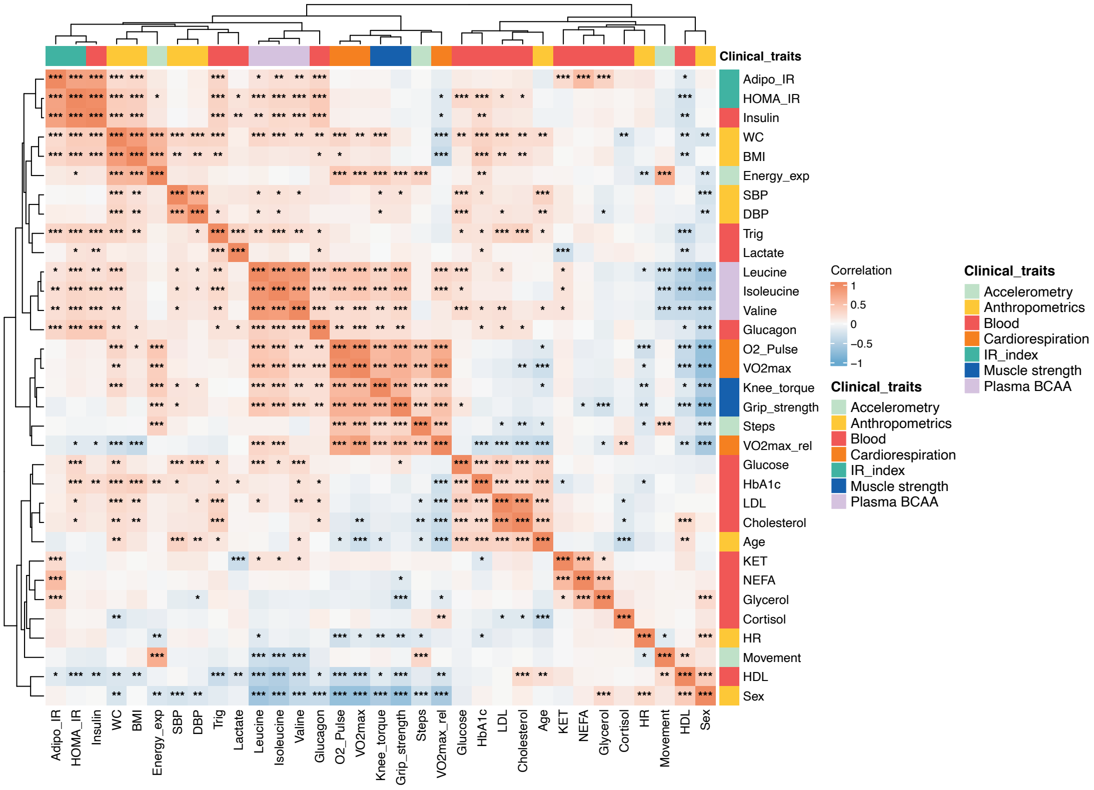

Fig S1B

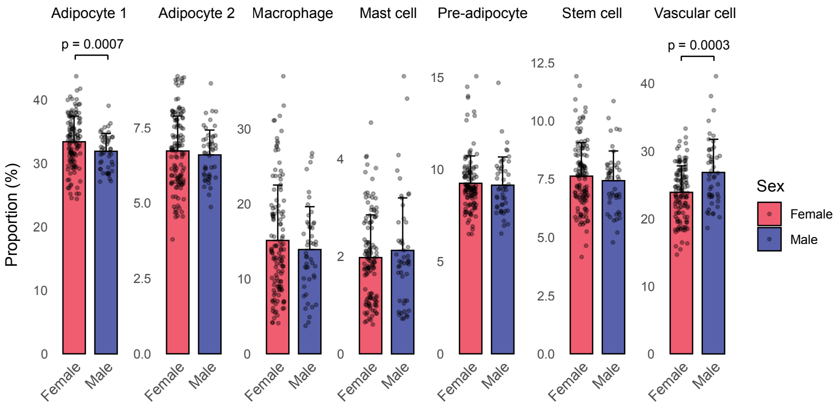

Figure S1. Intercorrelation of clinical/sub-clinical variables and sexual dimorphism in ASAT cell type proportions at baseline (Supplement to Figure 1)

A) Intercorrelation heatmap across clinical and subclinical variables. “p<0.05, **p<0.01, ***p<0.001. B) Sex-based comparisons of deconvoluted ASAT cell type proportions. BMI, Body mass index; WC, Waist circumference; SBP, Systolic blood pressure; DBP, DIastolic blood pressure; HR, Heart rate; NEFA, Non-esterifed fatty acid; Total_C, Total cholesterol; HDL, High-density lipoprotein; LDL, Low-density lipoprotein.

bioRxiv preprint doi: https://doi.org/10.64898/2026.03.05.702363; this version posted March 19, 2026. The copyright holder for this preprint (which was not certified by peer review) is the author/funder, who has granted bioRxiv a license to display the preprint in perpetuity. It is made available under aCC-BY 4.0 International license.

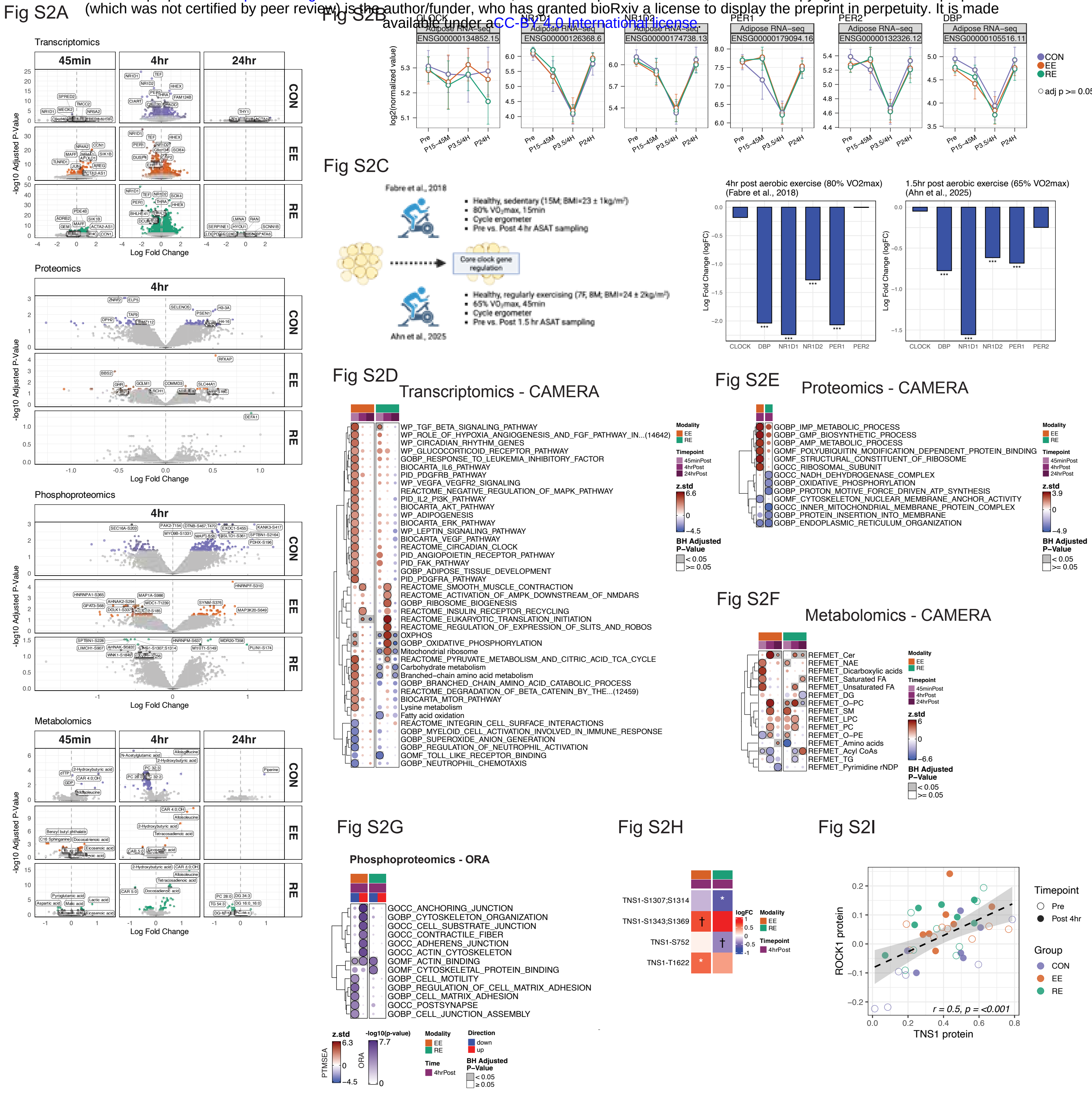

Figure S2. Differential analysis and enrichment analysis (Supplement to Figure 2)

A) Volcano plots showing differentially regulated features across omic layers and timepoints in CON, and in CON-unadjusted EE and RE groups. B) Temporal expression patterns of core circadian transcripts (CLOCK, NR1D1/2, and PER1/2, and DBP) in response to exercise. C) Acute exercise effects on core clock genes from previous reports (Fabre et al., 2018 and Ahn et al., 2025). In Fabre et al., 15 sedentary males without obesity performed 15min of moderate intensity endurance exercise (80% VO_2 max) on a cycle ergometer. ASAT sampling occurred 4 hr post-exercise. In Ahn et al., 15 regular exercisers (7 females and 8 males) without obesity performed 45min of moderate intensity endurance exercise (65% VO_2 max) on a cycle ergometer. ASAT sampling occurred 1.5 hr post-exercise. In both studies, ASAT transcriptome was profiles via bulk RNA sequencing. Bar plots show changes in core clock genes post-exercise in two studies. *adjusted p<0.05, **adjusted p<0.01, ***adjusted p<0.001. D) CAMERA-PR analysis of transcriptomic, E) proteomic, and F) metabolomic changes post-exercise. G) ORA results on collapsed differentially regulated phosphoproteins, stratified by directionality. H) Heatmap illustrating exercise responses on TNS1 phosphorylation at post 4 hr. *adjusted p<0.05, †adjusted p<0.1. I) Spearman correlation between protein abundance of ROCK1 and TNS1.

Fig S3

bioRxiv preprint doi: https://doi.org/10.64898/2026.03.05.702363; this version posted March 19, 2026. The copyright holder for this preprint (which was not certified by peer review) is the author/funder, who has granted bioRxiv a license to display the preprint in perpetuity. It is made available under aCC-BY 4.0 International license.

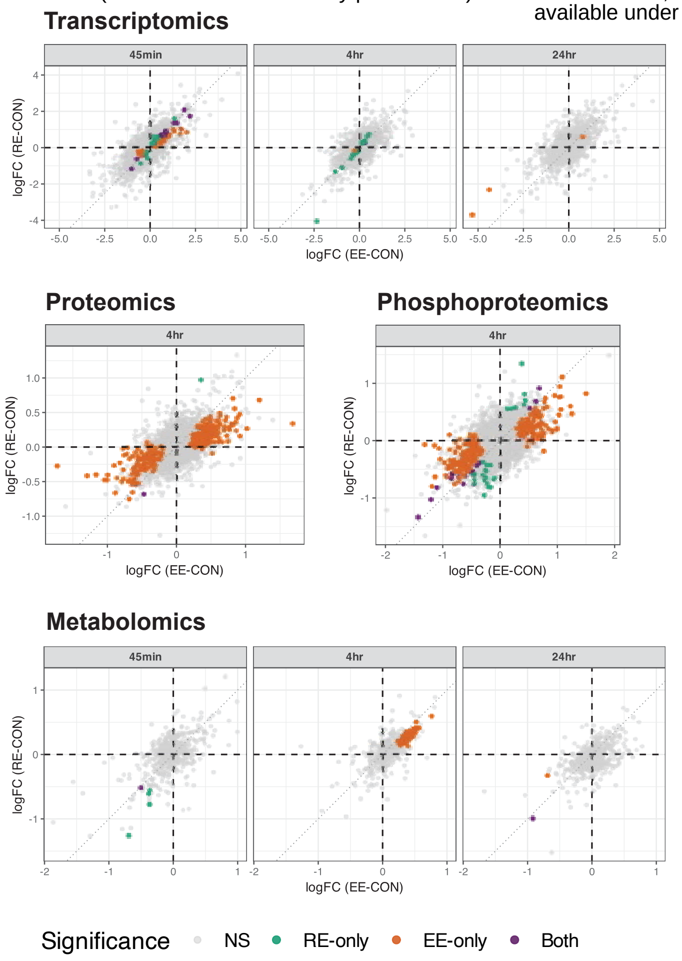

Figure S3. Comparison of EE vs. RE (Supplementary to Figure 3)
Scatter plots comparing logFC between EE and RE for features differentially regulated (adjusted p<0.05) in EE or RE.d p<0.05.

bioRxiv preprint doi: https://doi.org/10.64898/2026.03.05.702363; this version posted March 19, 2026. The copyright holder for this preprint (which was not certified by peer review) is the author/funder, who has granted bioRxiv a license to display the preprint in perpetuity. It is made available under aCC-BY 4.0 International license.

Fig S4A

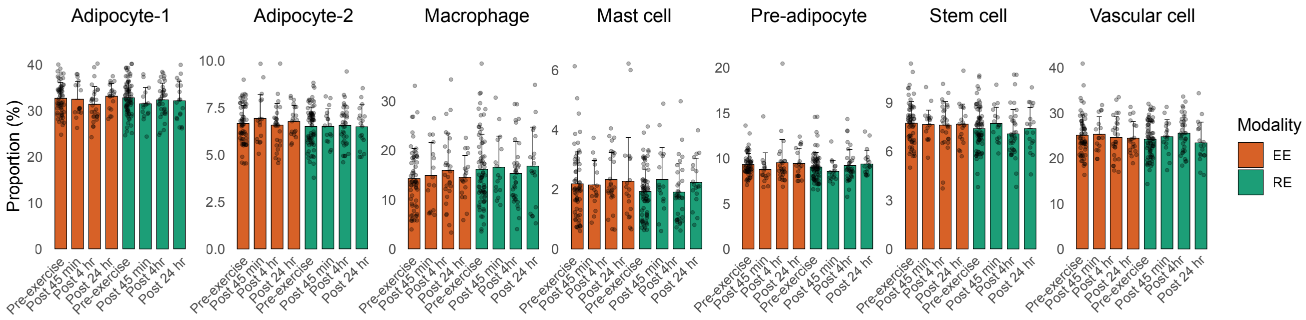

Fig S4B

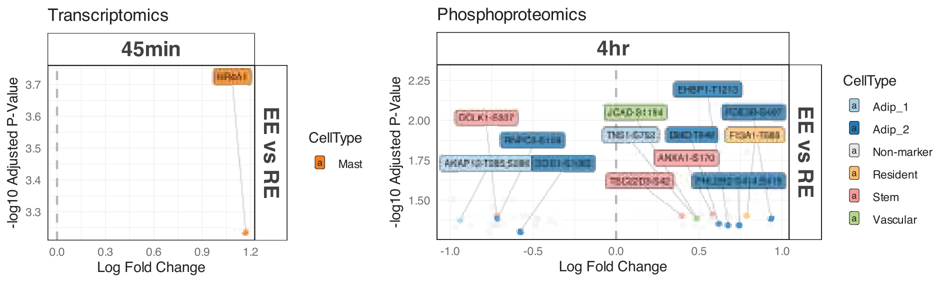

Fig S4C

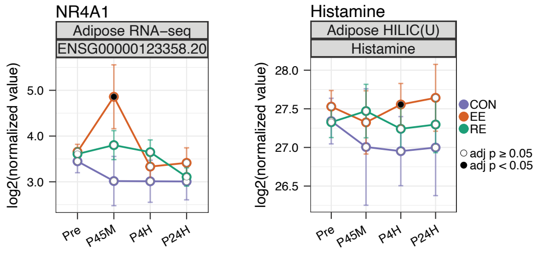

Figure S4. Cell type deconvolution and enrichment (Supplement to Figure 4)
A) Estimated ASAT cell type proportions (%) from deconvolution analysis at each timepoint, separated by exercise modality. No significant modality × timepoint interaction or main effect was detected. B) Volcano plots of differentially regulated phosphosites in EE vs. RE, focusing on features annotated as cell-type markers. logFC > 0 indicates greater enrichment in EE; logFC < 0 indicates greater enrichment in RE. C) Temporal expression patterns of *NR4A1* and histamine in ASAT.

bioRxiv preprint doi: https://doi.org/10.64898/2026.03.05.702363; this version posted March 19, 2026. The copyright holder for this preprint (which was not certified by peer review) is the author/funder, who has granted bioRxiv a license to display the preprint in perpetuity. It is made available under aCC-BY 4.0 International license.

Fig S5A

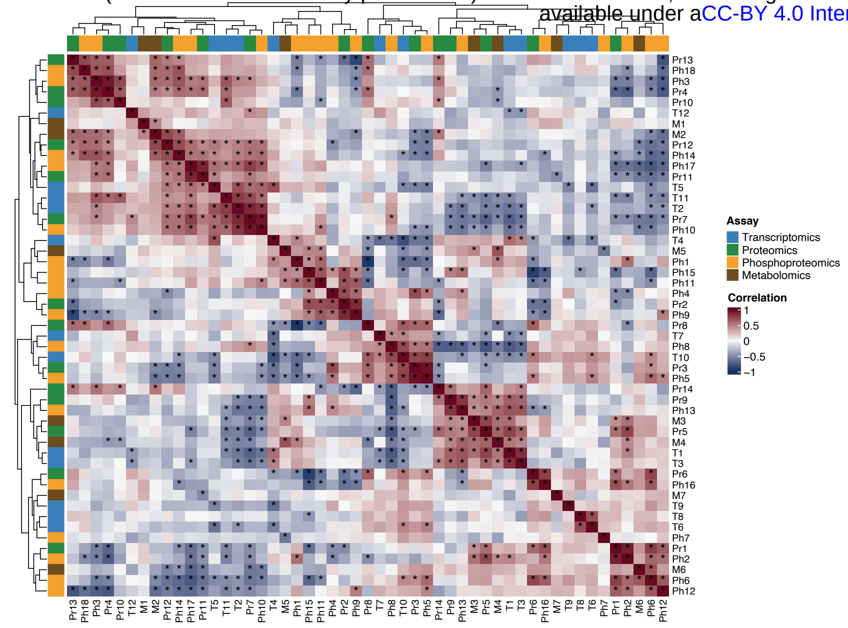

Fig S5B

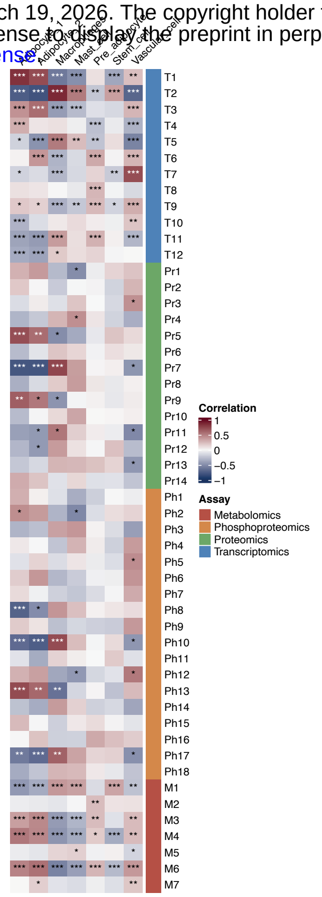

Fig S5C

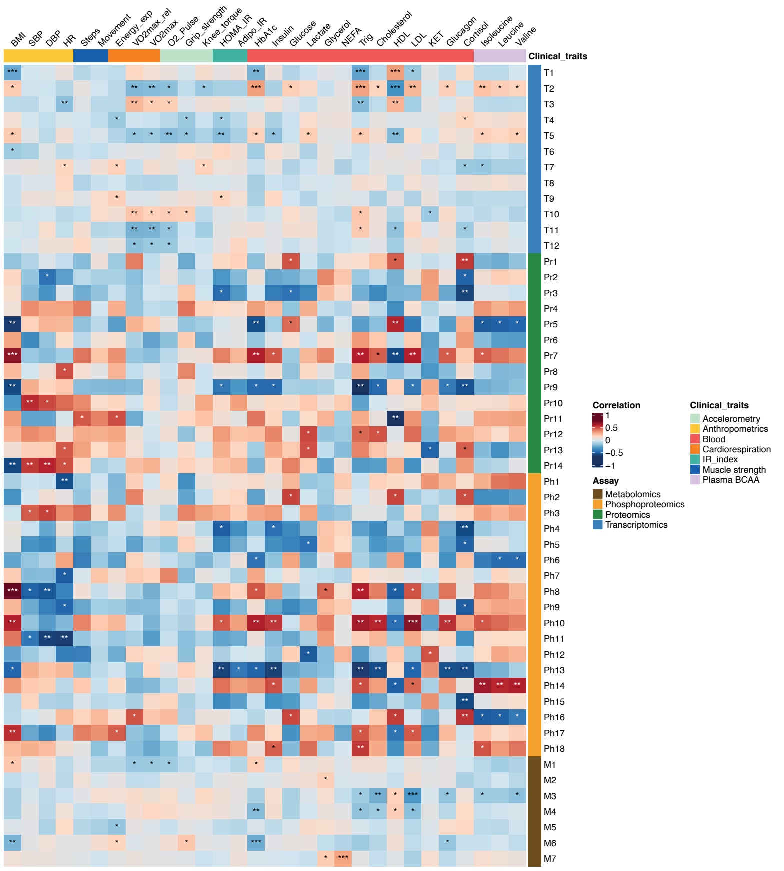

Figure S5. Integrative network downstream correlation analyses (Supplement to Figure 5)

A) Heatmap illustrating cross-omic correlation of constructed modules. *p<0.05. B) Heatmap showing unadjusted biweight midcorrelations between module eigengenes and estimated ASAT cell type proportions from baseline deconvolution. *p<0.05; **p<0.01; ***p<0.001. C) Heatmap showing biweight midcorrelations between module eigengenes and clinical traits, adjusted for sex, age group, and waist circumference (WC). Covariate adjustment was performed by regressing both traits and eigengenes, and residuals were used for correlation. *p<0.05; **p<0.01; ***p<0.001.

bioRxiv preprint doi: https://doi.org/10.64898/2026.03.05.702363; this version posted March 19, 2026. The copyright holder for this preprint (which was not certified by peer review) is the author/funder, who has granted bioRxiv a license to display the preprint in perpetuity. It is made available under aCC-BY 4.0 International license.

Fig S6A

Fig S6B

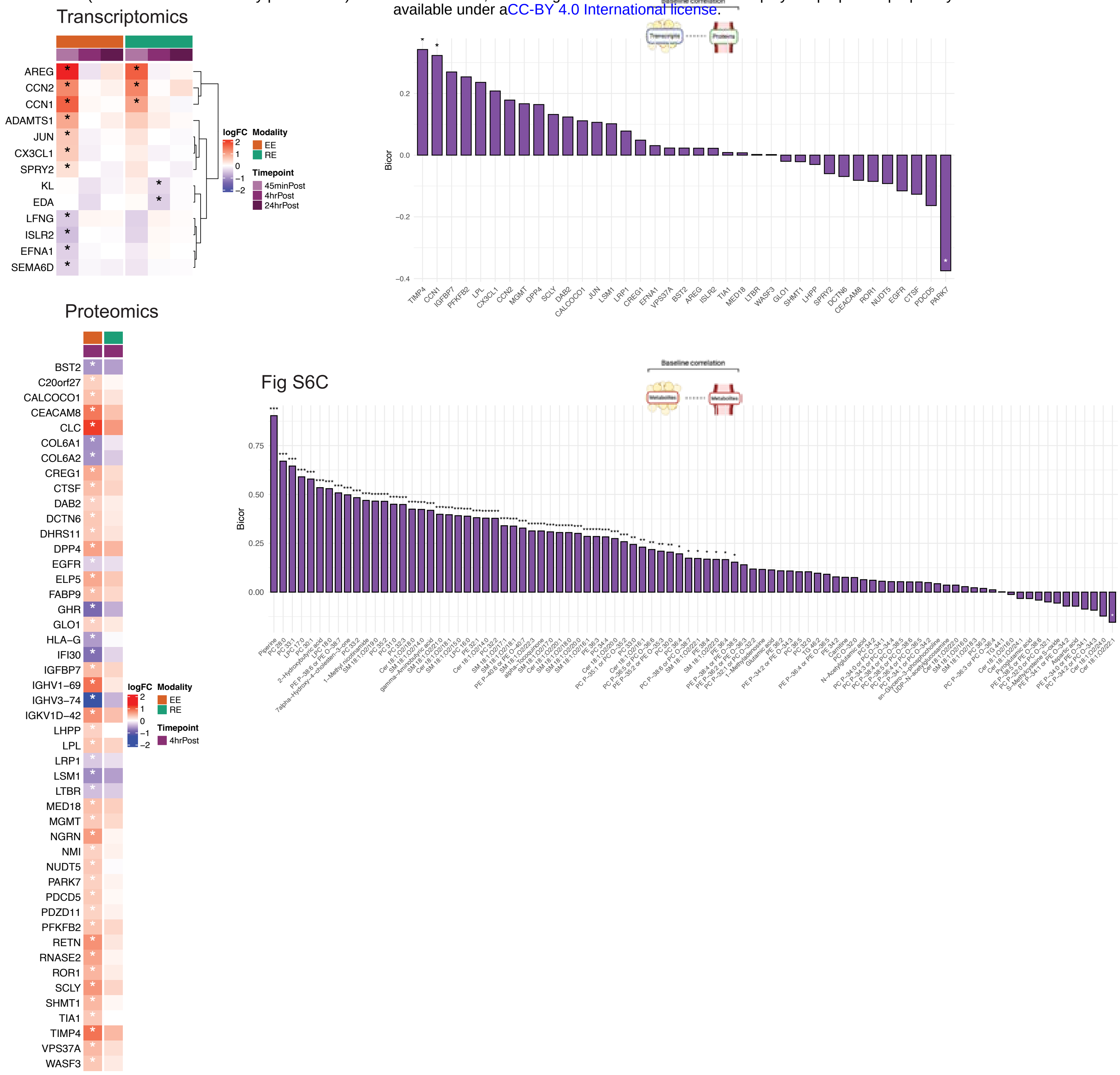

Figure S6. Relationship between ASAT feature abundance and corresponding plasma feature abundance at baseline (Supplementary to Figure 6)

A) Heatmaps illustrating exercise responses on filtered exerkine candidates. *adjusted p<0.05. B) Biweight midcorrelation between ASAT-exerkine transcript expression and corresponding plasma protein abundance at baseline. *p<0.05. C) Biweight midcorrelation between ASAT-exerkine metabolites and corresponding plasma metabolites at baseline. *p<0.05, **p<0.01, ***p<0.001

bioRxiv preprint doi: https://doi.org/10.64898/2026.03.05.702363; this version posted March 19, 2026. The copyright holder for this preprint (which was not certified by peer review) is the author/funder, who has granted bioRxiv a license to display the preprint in perpetuity. It is made available under aCC-BY 4.0 International license.

Fig S7A

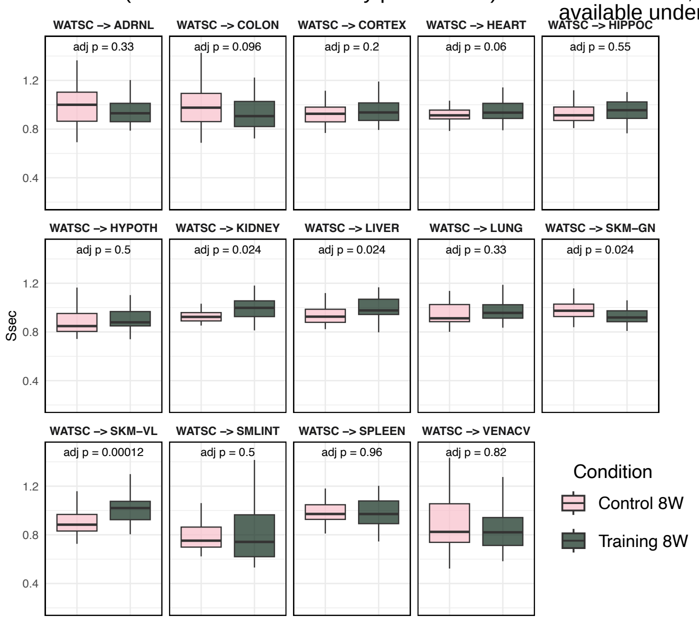

Fig S7B

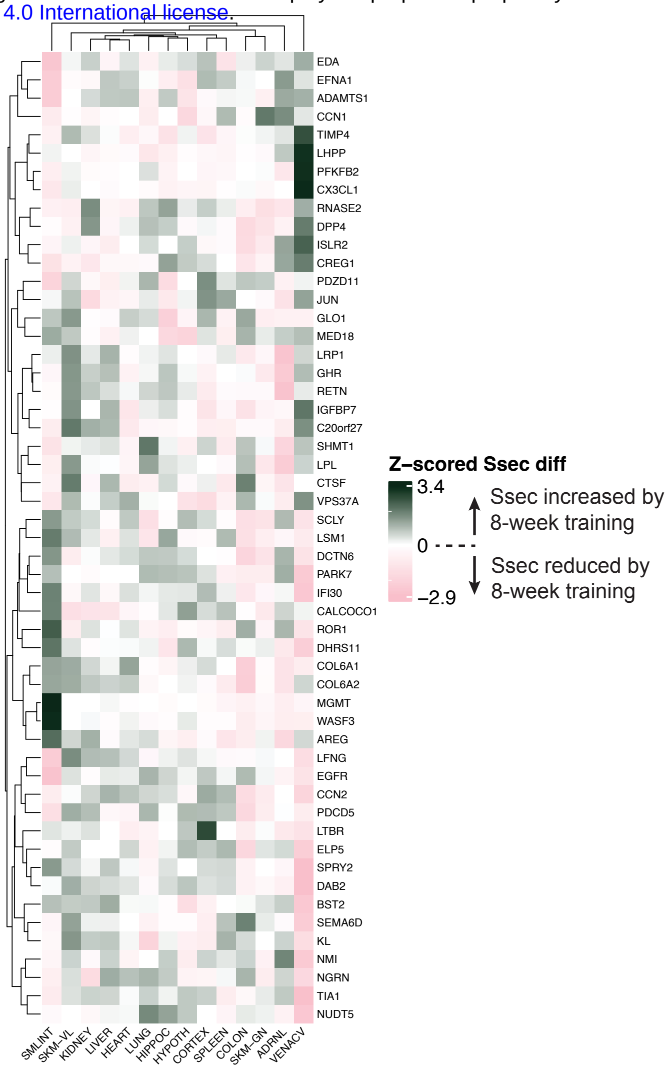

Figure S7. ASAT-exerkine candidates in endurance training (Supplementary to Figure 7)

A) Paired T-test results comparing Ssec for each WATSC-to-target tissue connection between CON and TR8W. Pink boxplot refers to CON. Dark green boxplot refers to TR8W. B) Heatmap showing Ssec difference of 60 ASAT-secreted candidates between TR8W vs. CON. Positive z-scaled Ssec diff indicates higher Ssec observed in the 8-week trained rats compared with 8-week untrained rats for the given WATSC-to-target tissue connection.
